# Hogness at one hundred

**DOI:** 10.1093/genetics/iyaf171

**Published:** 2025-11-12

**Authors:** David M Kingsley, William S Talbot

**Affiliations:** Howard Hughes Medical Institute and Department of Developmental Biology, Stanford, CA 94305, United States; Department of Developmental Biology, Stanford University School of Medicine, Stanford University, Stanford, CA 94305, United States

**Keywords:** genomics, molecular genetics, chromosome walking, *Escherichia coli*, *lac* operon, lambda phage, *Drosophila*, development, homeotic genes

## Abstract

During his remarkable scientific career, David S. Hogness transformed the molecular analysis of genes, genomes, and animal development. Hogness was born 1 century ago this month, on November 17, 1925. On the 100th anniversary of his birth, we would like to share an autobiographical account that Hogness wrote in 1992, 7 years before he retired.

## Teaser

During his remarkable scientific career, David S. Hogness transformed the molecular analysis of genes, genomes, and animal development. Hogness was born one century ago this month, on November 17, 1925. On the hundredth anniversary of his birth, Kingsley and Talbot would like to share an autobiographical account that Hogness wrote in 1992, seven years before he retired.

“when you do science, you potentially change the world much more than Caesar or any of the great military or political figures ever did, and you can sit very quietly in a corner and do that.”

– Max Delbrück 1978

David [“Dave”] S. Hogness was born 1 century ago this month, on November 17, 1925. During his remarkable scientific career, Dave transformed the molecular analysis of genes, genomes, and animal development.

His work spanned multiple topics in different organisms, including gene regulation in *Escherichia coli* as a postdoc with Jacques Monod in Paris (1952 to 1954), colinearity between traits and DNA features in phage lambda as a faculty member first at Washington University and then at Stanford University (1955 to 1968), and pioneering the molecular analysis of animal development in *Drosophila* (1968 to 1999). At every stage of his career, Dave chose fundamental biological problems and developed innovative molecular methods to solve them. He had a remarkable ability to think across many different scales. He also had a fearless optimism that grand biological mysteries could be solved by applying tools of biochemistry and molecular biology. His studies showed how genetically defined traits in any organism could be traced to genes and mutations, and how whole genomes could be cloned, fractionated, and reassembled in ways that became the foundation for modern genomics.

**Fig. 1.**
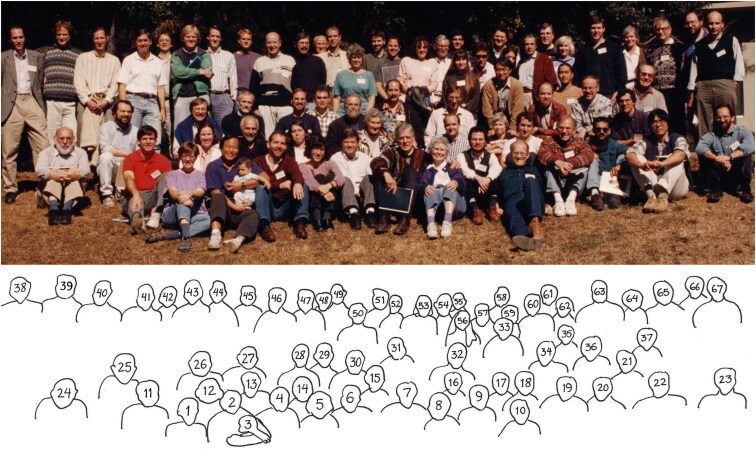
(Top) Group photograph of participants at “From Phage to Flies: Forty Years of Discovery”, a symposium held in October 1995 in honor of Dave Hogness' seventieth birthday. (Bottom) Key to participants' names. Dave and Judy Hogness are seated near the center of the front row. Row 1: (sitting on ground): 1. Anne Villeneuve, 2. Stuart Kim, 3. Jesse Kim, 4. Michael Bender, 5. Liz Gavis, 6. Steve Helfand, 7. Dave Hogness, 8. Judy Hogness, 9. Howard Lipshitz, 10. Ken Burtis; Row 2: 11. Scot Munroe, 12. Karen Telander-Muskavitch, 13. Fotis Kafatos, 14. Mariana Wolfner, 15. Annamaria Torriani-Gorini, 16. Mike Koelle, 17. Lynn Riddiford, 18. Ken Irvine, 19. Michael Grunstein, 20. Sanjay Jha, 21. Carl Thummel, 22. Shige Sakonju, 23. Mark Krasnow; Row 3: 24. Walter Gehring, 25. Fred Schachat, 26. Marc Telander Muskavitch, 27. Jim Champoux, 28. Peter Harte, 29. Rob Saint, 30. Michael Ashburner, 31. Elliot Meyerowitz, 32. George Ordal, 33. Toshi Watanabe, 34. Jim Truman, 35. Pat Hurban, 36. John Lis, 37. Mel Cohn; Row 5: (standing): 38. Jeremy Nathans, 39. Dan Garza, 40. Kerry Kornfeld, 41. Rick Lifton, 42. Phil Beachy, 43. Mike Young, 44. David Kingsley, 45. John Hiebert, 46. Walter Doerfler, 47. Carl Parker, 48. Alfred Tissieres, 49. David Glover, 50. Anne Baldwin, 51. Will Talbot, 52. Richard Mann, 53. Suzanne Bourgeois, 54. Buzz Baldwin, 55. Farhad Imam, 56. Michelle Arbeitman, 57. Steve Stowers, 58. John Carlson, 59. Popi Artavantis-Tsakonas (partly hidden), 60. Spyros Artavanis-Tsakonas, 61. Mark Pearson, 62. Kathy Pearson, 63. Kevin White, 64. Barbara Hobom, 65. Gerd Hobom, 66. Gerry Rubin, and 67. David Finnegan. Photograph and caption information from Hogness papers, Special Collections and University Archives, Stanford University Libraries.

In addition to a long chain of major advances from his own lab, Dave also inspired an entire generation of researchers to apply molecular methods to other systems. For example, after studying *Drosophila* molecular genetics with Dave as a graduate student, one of the current authors (W.S.T.) applied Hogness-inspired positional cloning approaches to the study of mutations that disrupt zebrafish development. After hearing a research seminar by Dave in graduate school, DMK was inspired to use positional cloning methods to isolate mutant genes controlling classical skeletal traits in mice, and then major loci controlling evolutionary differences in wild stickleback fish. Similar stories could be told by countless other scientists. The academic tree website has a partial listing of the truly remarkable set of students and postdocs who trained with Dave Hogness, and who then pioneered molecular studies of a whole range of biological phenomena in diverse systems (Flytree 2025). Many of these trainees and collaborators gathered for a science symposium and celebration when Dave was turning 70 in 1995. The picture from this event helps capture just how many scientific leaders in *Drosophila* and other fields can trace their scientific roots to the Hogness Lab (Fig. 1).

Many of Dave's scientific accomplishments have previously been well summarized in biographical sketches, memorials, and award citations, including receipt of both the GSA Medal (1984) and the Thomas Hunt Morgan Medal (2003) from the Genetics Society of America (e.g. Burtis et al. 2003; Young et al. 2024). On the 100th anniversary of his birth, we would like to share the following autobiographical account that Dave wrote 7 yr before he retired. We have added a few explanatory notes, shown in brackets, and the opening quotation by Max Delbrück (Delbrück 1978). Max had an important influence on Dave's career, and Dave Hogness did go on to change the world with his own quiet thinking and remarkable research.

—————————————

Ascona, May 26, 1992. [Dave faxed the draft of this essay to Stanford (where it was overdue at the time), during a famous 1992 EMBO Workshop on “The Homeobox and the Genetic Control of Development” in Ascona, Switzerland. Most of the meeting celebrated the remarkable advances in developmental biology that had followed soon after his laboratory's first positional cloning of a *Drosophila* homeotic gene. Meanwhile Dave's own laboratory had begun to focus on additional genetic hierarchies that govern developmental timing.]

Thoughts on Becoming the First Holder of the Rudy J. and Daphne Donohue Munzer Professorship in the School of Medicine

“Medicine takes its lessons from an ever-increasing array of disciplines. The intangible and complex constructs of computer science are grist for its mill, as are the most recent concepts in the biological sciences. Indeed, there is little that does not feed medicine's appetite for improvement. Perhaps, the astrophysics of the Big Bang is beyond assimilation, but the applications of nuclear magnetic resonance have had their obvious impact both in clinical analysis and in the determination of the structure and function of proteins that are so important to our health. In the near future, we should have at our computer-educated fingertips the DNA sequences of the entire human genome and be faced with the awesome puzzle of what to do with that information.

I note this extraordinary expansion of medicine's base to emphasize that one would be hard put to guess what the holder of the Munzer Professorship actually did, or had done, given only that it is a Professorship in the School of Medicine. Certainly, my background would not have helped the guesser, for my first career goal was to be either a physicist or a Supreme Court justice. Even during my undergraduate and graduate days at Caltech, when I shifted my interests from physics and chemistry to chemical genetics (the precursor of today's molecular genetics)—even then I had no interest or inclination to join the faculty of a school of medicine.

A major transition in my scientific interests occurred near the end of my graduate years (1949 to 1952) toward a Ph.D. in Chemistry. Max Delbrück had asked me to present to his phage group in Caltech's Biology Division a manuscript he had received from Jacques Monod in which he and Melvin Cohn presented a historic analysis of the problem of induced enzyme synthesis in bacteria (Monod and Cohn 1952). [In a handwritten note on his reprint of this paper, Dave states “Reviewed for Delbrück et al. phage group”.] I was fascinated by this problem of how the rate of synthesis of a specific protein could be increased over a 1000-fold by the addition of a small molecule, the inducer. I therefore resolved to carry out my postdoctoral research in Jacques' laboratory at the Institut Pasteur in Paris, arriving there in 1952.

Looking back, I view this move as my first critical step into developmental biology, or more specifically, into the molecular genetics of development. Although *E. coli*, the bacterium used for studying induced enzyme synthesis, would not normally qualify for developmental studies, induced enzyme synthesis provided one of the earliest and best models of how gene activity is regulated at the molecular level, and genetic regulation is basic to the developmental processes by which the adult organism is formed from the fertilized egg.

When I joined Jacques' laboratory, however, our understanding of the molecular mechanisms by which genetic activity is regulated was virtually nonexistent, nor did we then *know* that induced enzyme synthesis provided an example of that regulation. Hence, the questions we asked were quite basic. In the case at hand, for example, we knew only that the enzyme activity increased dramatically after addition of the inducer; we did not, however, know whether that increase in activity resulted from the de novo synthesis of the protein providing that activity from its constituent amino acids, or resulted from some other mechanism such as an inducer-directed conversion of an inactive precursor protein into the active enzyme. If the first proposition were true, then it was clear that induced enzyme synthesis would provide a beautiful system for studying genetic regulation, whereas if the second were true, it would not.

Mel Cohn and I worked together on this basic problem in Jacques' laboratory, providing the first proof that induced enzyme synthesis did indeed represent de novo protein synthesis. The induced synthesis of the enzyme that was the focus of our studies, and of Jacques' laboratory generally, β-galactosidase, thereafter did provide a beautiful system for studying genetic regulation—in this instance, the regulation of the *lac Z* gene by the inducers that led to Jacques' Nobel Prize several years later. For me, those 2 yr in Paris were not only formative but they were among the most exhilarating I have known. We were all young, brash, and knew we were doing important things. No one asked whether this or that was worth doing, we simply knew it was so.

On returning to the United States, I was invited to join the Department of Microbiology that Arthur Kornberg was developing at Washington University School of Medicine. This was the first time the idea of joining a medical school had been broached in serious terms, and I must admit I found the idea foreign and strange. I had also been invited to join Yale's Chemistry Department by Jack Kirkwood, its chairman, and that was more within the context of my preconceptions. Upon closer examination, however, it turned out that Yale's Chemistry was foreign and Washington's Microbiology was familiar. I had obviously crossed the Rubicon. Arthur had already collected a cast of characters which was irresistible, including as [it] did Mel Cohn, Paul Berg, and Bob Lehman.

Dale Kaiser joined the department shortly after I arrived, marking the third recruit from the lnstitut Pasteur, where he had worked with François Jacob and André Lwoff. There I began working on lambda, in collaboration with Dale. Our aim was to develop a system in which the naked lambda DNA would infect *E. coli.* Together we used the system to prove that the physical location of the genes in the lambda DNA molecule is colinear with their position on the recombination map. Recombination mapping had been invented by Sturtevant to map the position of genes in the *Drosophila* chromosomes some 40 yr earlier, and while it had been postulated that such a recombination map gave the order of the genes in the chromosomal DNA, our work provided the first proof of that postulate. During this period, the entire department moved to Stanford in 1959, to become Stanford's first Department of Biochemistry, again within a School of Medicine.

After I had spent a dozen years working chiefly on the developmental genetics of lambda, this bacterial virus had become such a focus of attention that the field became overcrowded. Indeed, I once calculated that there were so many laboratories working on lambda DNA (which is 50,000 base pairs long) that there was 1 laboratory for every kilobase pair. To appreciate this concentration, if there were a similar density of molecular geneticists working on the human genome, it would require 3,000,000 laboratories, each with a team of 10, or about 30 million scientists.

Not only was lambda overcrowded, but I felt another constraint, namely, there was little to argue over as the experimental routes to answering most questions were fairly obvious. I therefore decided in 1968 that it was time to move upward to more complex organisms where almost nothing was known about the molecular genetics of development, where experimental routes toward expanding such knowledge had not been devised, and where argument was rife. In short, I decided to enter the classical field of developmental biology with the view of declassifying it.

The first question was what organism would be most tractable. I had pretty much settled on the fruit fly, *Drosophila* melanogaster, for 3 reasons: (i) its well developed and efficient system for genetic analysis, (ii) its giant polytene chromosomes, which provided the best physical map of genetic loci among higher organisms, and (iii) its relatively small genome, which I had calculated from Rudkin's data contained 170,000 kilobase pairs, about 3,400 times larger than lambda, but almost 20-fold smaller than the human genome. But as I was not sure that *Drosophila* was the organism of choice, I took my first sabbatical in 1968–1969 to learn more. The first of its 3 parts was spent at Caltech in the laboratory of Ed Lewis, who had been working since 1946 on *Drosophila* homeotic mutations that identified genes which I thought the most interesting in developmental genetics. These mutations yielded flies that have an extra pair of wings, or an extra pair of legs, etc., indicating that the normal function of the respective homeotic genes was to decide where such structures as legs and wings, and even eyes and antennae, should be made! The next third was spent with Jim Peacock, a brash young Australian geneticist who knew about everything, not only flies, but also kangaroos, which I had considered as a possible choice because of their small number of chromosomes. Jim was also receptive to new ideas and approaches that could open developmental biology to molecular genetic analysis. [Though kangaroos seem unrelated, during his visit Dave and Peacock considered strategies to purify *Drosophila* chromosomes from millions of synchronized colchicine-treated blastoderm embryos.] The final third was spent in the Max Planck Institute in Tübingen working with Wolfgang Beerman, who knew more about polytene chromosomes than anyone else.

This sabbatical marked another critical time in my research. Like the first, it was for me a time of great excitement and enthusiasm. Unlike the first, however, my plans to analyze *Drosophila* genes at the molecular level, with the eventual aim of understanding the homeotic genes at that level, met with much skepticism—most of my friends thinking that I was a bit crazy. On objective analysis, I should probably have thought so as well, but for reasons I cannot quite comprehend, I again had no doubts that I was on the right track, sufficiently so that upon returning from sabbatical I immediately transformed my laboratory from lambda to *Drosophila*.

The work on *Drosophila* went predictably slowly at first, but by 1973 we were hard at work trying to make recombinant DNA molecules between *Drosophila* and lambda vector DNAs, using ideas that were floating around the department at that time, chiefly in the laboratories of Paul Berg, Ron Davis and Dale Kaiser, as well as our own. By 1974 we had made libraries of cloned *Drosophila* DNA segments, now using a plasmid vector, and were able to identify the chromosomal loci from which individual cloned segments derived by hybridization of tritium-labeled cloned DNA to the polytene chromosomes in situ.

Thereafter, the work moved at a rapid pace. The development of the new techniques that we needed to identify cloned DNAs from particular genes came with seemingly, in retrospect, effortless ease. Thus, we invented colony hybridization to identify cloned DNAs containing a given sequence, which, for example, allowed us to identify clones containing genes that produced a given RNA, such as the ribosomal RNA genes, the histone genes and the heat shock genes (see also accompanying paper by Restifo et al. 2025). The pace quickened further by the development of additional techniques from other laboratories, chiefly that of Ron Davis. Clearly, we were in the right place at the right time.

We were still, however, lacking the techniques we needed for the isolation of the most interesting genes from the viewpoint of a developmental geneticist, which for me meant the *Drosophila* homeotic genes. This lack derived from the fact that these genes were defined solely by their mutations, and their positions on genetic recombination maps or in the polytene chromosomes. Their RNA and protein products were unknown, and existing techniques for clonal identification relied upon a knowledge of these products, or upon DNA sequences that were, of course, also unknown for the homeotic genes.

We therefore invented 2 new techniques that provided the means for isolating virtually any gene in *Drosophila* for which mutations had been identified. These we called “chromosomal walking” and “chromosomal jumping,” and by their use we identified cloned DNA from a homeotic gene called *Ultrabithorax* in 1979, some 10 yr after my initiating sabbatical. This gene was molecularly identified and defined initially by mapping the sites of its mutations in the cloned DNAs, and subsequently by identifying the corresponding transcription unit and its messenger RNAs from which the *Ultrabithorax* proteins were then identified.

This brings me to 1980, the year when the explosion in the molecular genetics of development really began, for we not only had in hand the techniques for isolating any gene defined by mutation, but 1980 marks the year that Eric Wieschaus and Christiane Nüsslein–Volhard reported on their identification by mutation of a panoply of genes that control the embryonic development of *Drosophila* and thereby set up most of the patterns for the development of the adult fly. That explosion has carried into other animals, including man, and most recently into plants. The carrier for these extensions is not only the assorted techniques noted above, but also the unexpected evolutionary conservation of the genetic elements critical to the development of species so widely separated as *Drosophila* and man.

The impact of this explosion was felt locally so that by the mid-1980s when plans for the Beckman Center were initiated, it became obvious that one of the units it should house was a new Department of Developmental Biology. I became involved in the effort to create such a department by chairing a select committee whose first task was to define the nature of this department. We were to take into account not only the explosive nature of the field, but also the context of the Beckman Center with its mission of extending such bursts of biological knowledge to clinical applications in the simplest and most efficient manner. Having accomplished that first task, we began the second task of finding a chair who would develop such a unique department dedicated solely to developmental biology. Here, we had the great good fortune of attracting Lucy Shapiro to that position, for during the past few years she has fashioned an extraordinary group of developmental biologists who form the unique department that we envisaged, and which I have had the good fortune of joining.

I note my role in creating this department because of its relevance to the Munzers' interest in the Beckman Center and, I suppose, my selection as the first holder of the Munzer Professorship. I view the unexpected honor of being chosen as the first Rudy J. and Daphne Donohue Munzer Professor both as a mark of things past and as a vote of confidence not only in my future but also in the future of this new and unique Department of Developmental Biology. In my eyes, its several members are as worthy as I, if not more so, of the honor of such a professorship—an honor that I trust will be theirs 1 day. [As Dave predicted, the Munzer chair is currently held by David Kingsley in the same Developmental Biology department that Hogness helped to create.] As for me, the field of homeotic gene research has, unbelievably, become crowded, and I have moved on to investigate another set of genes that coordinate the developmental pathways of different tissues under the aegis of a steroid hormone.”

—————————————

While Dave's research summary ends there, during the same week he was preparing this retrospective, he also presented a keynote presentation at Ascona on “Temporal coordination of *Drosophila* development by genetic regulatory hierarchies that respond to ecdysone”. In his talk, Dave posited that “the molecular genetic analysis of timing has reached the same level of respectability as that for spatial patterning.” He presented his group's research on the discovery of the ecdysone receptor that regulates a hierarchy of target genes and tissue responses during insect metamorphosis. Like homeotic genes, members of the steroid receptor superfamily are conserved across diverse animals, and their discovery revealed ancient molecular relationships between metamorphosis pathways in insects, frogs, and mammals.

After retiring in 1999, Dave continued to receive awards for his pioneering studies. In addition to the Thomas Hunt Morgan Medal (Burtis et al. 2003), Dave was recognized by the Warren Alpert Foundation Prize for helping to lay the groundwork for human genetics (Warren Alpert Foundation 2013) and (International Prize in Biology in 2007). Now in 2025, on the 100th anniversary of his birth, it seems fitting to conclude with some remarks Dave delivered at the 2007 symposium honoring his lifetime of contributions in biology.

“In the years since I first entered the field, much has been learned about the genetic control of development. I am glad that I have been able to add to our shared knowledge in this area, in the understanding of the regulation of gene expression in *Drosophila melanogaster*, and in bringing the analysis of gene expression and development to the molecular level. Over these decades, the field has undergone considerable changes, in step with changes in the tools of science. Today computers play a role in developmental biology that we take for granted. In sequencing, for example, computers can assemble sequences and help analyze the results, allowing us to get a handle on vast quantities of data that would be difficult or impossible for any one person to hold in their brain at one time.

This brings to mind a story about the first president of Stanford University, David Starr Jordan–who like the Emperor, was an ichthyologist. [The Emperor of Japan was in the audience for this address.] When Stanford first opened, Jordan was known for remembering the name of every new student, and greeting each one by name as he walked across campus. But he stopped doing this after a few years. When asked why, Jordan answered that after a few years he found that every time he memorized the name of a new student, he forgot the name of a fish. “It was a bad bargain,” Jordan said.

Perhaps the real lesson is that the work of science can never be the work of just one person's mind that our most important knowledge is always shared knowledge, where one person's achievement stands on the shoulders of many others. And so I would like to thank all those in my laboratory who contributed to our research. In honoring me today, the Prize Committee is honoring them as well.

Thanks to you all.”
